# The Profile and Bioaccessibility of Phenolic Compounds in Cereals Influenced by Improved Extrusion Cooking Treatment

**DOI:** 10.1371/journal.pone.0161086

**Published:** 2016-08-11

**Authors:** Zicong Zeng, Chengmei Liu, Shunjing Luo, Jun Chen, Ersheng Gong

**Affiliations:** State Key Laboratory of Food Science and Technology, Nanchang University, No. 235 Nanjing East Road, Nanchang, Jiangxi, China; National Taiwan University, TAIWAN

## Abstract

The aim of this study was to investigate the effect of Improved Extrusion Cooking Treatment (IECT) on the phenolics and its bioaccessibility in cereals, represented by brown rice, wheat, and oat. Data showed that total phenolic content and total antioxidant activity in free form were significantly decreased, while the bound form was increased after IECT. After IECT, the total free phenolic acids of brown rice and wheat were significantly decreased by 5.88% and 45.66%, respectively, while the total bound phenolic acids of brown rice, wheat, and oat were significantly increased by 6.45%, 8.78%, and 9.10%, respectively. Brown rice provided the most bioaccessible phenolics and antioxidant compounds, followed by oat and wheat. IECT significantly decreased the bioaccessible phenolics of brown rice and oat by 31.09% and 30.95%, while it had minimal effect on the bioaccessible phenolics of wheat. These results showed that IECT greatly affected the phenolics and its bioaccessibiltiy of cereals, with the effect depending on cereal matrix and the sensitivity of free and bound phenolics. Furthermore, bioaccessible phenolic acids of raw and processed cereals were considerably low, and it slightly contributed to the bioaccessible phenolics.

## Introduction

Epidemiological studies have consistently shown that the consumption of whole cereals may contribute to the prevention of many chronic diseases, such as obesity, cardiovascular disease, type-2 diabetes, and some cancers [1]. The mechanisms behind the protective effect remain to be fully illuminated, but it is suggested that the health benefits are highly related to the phenolic compounds (phenolic acids and flavonoid) in cereals [2]. Phenolic compounds have two forms, namely free and bound forms, and bound phenolic compounds are referred to phenolic compounds which are covalently bound with cell wall components [1]. As the most common phenolic compounds in cereals, phenolics acids have strong antioxidant activity and may modulate cellular oxidative status and prevent biologically important molecules such as DNA, proteins, and membrane lipids from oxidative damage [3].

As consumers' awareness of health increases, there is an increasing demand for new processed foods of whole cereals, which is convenient and nutritious to satisfy the demands of health-conscious consumers. In our previous work, an Improved Extrusion Cooking Treatment (IECT) was reconstituted from traditional single-screw extruders. Compared to the traditional extrusion cooking machines, our transformed single-screw extruder had longer screw, longer residence time, higher die pressure, lower temperature and lower screw speed. Thus, the expansion of extrudate was hardly changed and the textured cereal (rice) made by IECT showed good texture properties [4]. However, the effect of this technique on the phenolic compounds in cereals was unknown. Therefore, it was important to determine the effect of IECT on free and bound phenolics in cereals.

In addition, previous studies focused on the effect of different processing technologies on phenolic content, antioxidant activity and phenolic profile of cereals. However, the total phenolic content and total antioxidant activity of cereals were probably underestimated, because most studies were based on the solvent extraction and quencher procedures which are both performed before digestion. The health potential of phenolics depended on their bioaccessibility, absorption in the gastrointestinal tract and their bioavailability *in vivo* [5]. Recent studies elucidated the effect of domestic processing [6–7], extrusion [8], and fermentation [9] on the bioaccessibility of phenolics in cereals. But the effect of IECT on the bioaccessibility of phenolics and antioxidant compounds was still unkown. Therefore, bioaccessibility was used to evaluate effect of IECT on phenolics in cereals.

In the present study, the effect of IECT on total phenolic content, total antioxidant activity and phenolic acids profile of cereals, represented by brown rice, wheat, and oat were evaluated. Especially, the bioaccessibility of phenolics and antioxidant compounds influenced by IECT were investigated after *in vitro* digestion.

## Materials and Methods

### Materials and Chemicals

Brown rice (*Oryza sativa L*.), wheat (*Triticum aestivum L*.) and oat (*Avena nuda L*.) were procured from local market. Standard phenolic acids (*p*-hydroxybenzoic acid, chlorogenic acid, vanillic acid, *trans*-caffeic acid, syringic acid, *trans*-*p*-coumaric acid, *trans*-ferulic acid, *trans*-sinapic acid) were procured from Sigma-Aldrich (St. Louis, MO, USA), and the *cis*-phenolic acids (*cis*-*p*-coumaric acid, *cis*-ferulic acid, *cis*-sinapic acid) were prepared by exposing a known solution of fresh *trans*-hydroxycinnamic acid to UV light overnight [10]. 2, 2' azino-bis (3-ethylbenzthiazoline) 6-sulfonic acid (ABTS), Folin-Ciocalteu reagent, digest enzymes (pepsin, pancreatin) were procured from Sigma-Aldrich (St. Louis, MO, USA). Bile extract (porcine origin) were procured from Solarbio (Beijing, China). HPLC-grade solvents were from Xilong (Guangdong, China). All other chemicals and reagents used were of analytical grade.

### Processing of Cereals by IECT

Cereals were ground and then processed by IECT according to Liu et al. [4]. The moisture content was approximately 30%, and the extrusion temperature was 120°C. Afterwards, samples were dried at 40°C, until the moisture content fell below 13%. Once cooled, all samples were transferred to polyethylene bags and stored at -20°C until used.

### Determination of Total Phenolic Content and Total Antioxidant Capacity

Extraction of phenolics was carried out according to Adom and Liu with slight modifications [11]. All of the samples were ground and screened through 60 mesh sieve. Free phenolics in cereals were extracted by blending one gram of sample with 20 mL of 80% chilled acetone for 10 min. After centrifugation at 2500 g for 10 min, the supernatant was removed and extraction was repeated. Supernatants were combined, evaporated at 45°C, and reconstituted with distilled water to a final volume of 25 mL. For the extraction of the bound phenolics, the residue was further hydrolyzed by 2 mol/L NaOH for 1 h with nitrogen flushing at room temperature. After alkaline hydrolysis, the mixture was neutralized with appropriate amount of HCl and extracted with hexane to remove lipids. The final solution was extracted five times with ethyl acetate, and then the ethyl acetate fraction was evaporated to dryness. Phenolics were reconstituted in 25 mL of distilled water. Free and bound extracts were kept at -20°C for further analysis of total phenolic content and total antioxidant activity.

Total phenolic content was determined using methods as described by Singleton et al. with slight modifications [12]. Briefly, the extracts (250 μL) were reacted with 250 μL Folin-Ciocalteu reagent for 6 min, then the reaction was neutralized with 2.5 mL 7% Na_2_CO_3_. The absorbance was measured at 760 nm after 90 min incubation. The measurement was compared with a standard curve of prepared ferulic acid solutions and expressed as micrograms of ferulic acid equivalents per gram dry weight sample (μg FAE/g DW).

The total antioxidant capacity was measured by ABTS radical scavenging capacity assay and expressed as μg Trolox/g DW [13].

### Determination of Phenolic Acid by HPLC

The crude extract of free phenolics was further extracted five times with ethyl acetate and then evaporated at 45°C. Both free and bound phenolics were dissolved in 5 mL of methanol/water (1:1 v/v), filtered, and injected into a RP-HPLC system. The content of phenolic acids was determined by HPLC system (Agilent 1260 Series) equipped with a variable-wavelength detector. Chromatographic separations were performed on a reverse phase C18 column (250 × 4.6 mm; 5 μm; SunFire, Waters, Massachusetts, USA) at 30°C. The mobile phase consisted of 1.3% glacial acetic acid in water (solvent A) and 100% acetonitrile (solvent B) with the gradient programme as follows: 0–2 min, 10% B; 2–20 min, 10–13% B; 20–35 min, 13–20% B; 35–45 min, 20–35% B; 45–80 min, 35–45% B; 80–93 min, 45–100% B; 93–98 min, 100–8% B; 98–105 min, 8% B. The sample (20 μL) was injected by auto-sampler, and the flow rate was maintained at 0.8 mL/min. Phenolic acids in the samples were detected at 280 nm, and they were identified on the basis of retention time and the chromatography of authentic standards. The electronic outputs of the detector were collected by ChemStation software (B.04.03, Agilent, USA) and were used to determine phenolic acids. The results were expressed as μg/g DW.

### In Vitro Bioaccessibility of Phenolic Compounds

To monitor the release of phenolics from the cereal matrix, an *in vitro* gastrointestinal digestion was performed as described by Mateos et al. with suitable modifications [14]. Initially, simulated gastric digestion was carried out by incubating a mixture of ground samples and pepsin (40 mg/mL in 0.1 mol/L HCl) at pH 2.0 in a shaking water bath at 37°C for 2 h. A blank was prepared with identical chemicals, and underwent the same conditions as the samples. To simulate intestinal digestion, segments of dialysis tubing (molecular mass cut off: 8000–12000 Da) filled with 5.5 mL 0.9% NaCl and 5.5 mL 0.5 mol/L NaHCO_3_ were immersed into the gastric digesta immediately after digestion. The amount of NaHCO_3_ was equivalent to titratable acidity of the gastric digest. The samples were then incubated in a shaking water bath at 37°C for 45 min till the pH of the digest was increased to ca. 6.5, reflecting the transition from the gastric phase to the intestinal phase. Then, 5 mL mixture of pancreatin-bile extract (4 mg/mL pancreatin and 25 mg/mL bile extract in 0.1 mol/L NaHCO_3_) was added to the digesta, and incubation was continued for 2 h till the pH of the digest reached ca. 7.0. At the end of simulated gastrointestinal digestion, the dialyzate was analyzed for total phenolic content, total antioxidant capacity, and phenolic acid as described above. The total phenolic content recovered in the dialysate samples after *in vitro* digestion was calculated and represented the bioaccessible phenolics. The percent bioaccessibility was calculated by the ratio of bioaccessible phenolics to the total phenolic content in cereals before digestion.

### Statistical Analysis

All determinations were made in triplicate, and the statistical analyses were performed using SPSS 17.0 (SPSS Inc., USA). Data are expressed as mean ± SD. Differences between raw and processed samples were calculated using Student’s t-test at a 5% significance level.

## Results and Discussion

### Effect of IECT on Total Phenolic Content and Total Antioxidant Activity of Cereals

**Fig 1:**
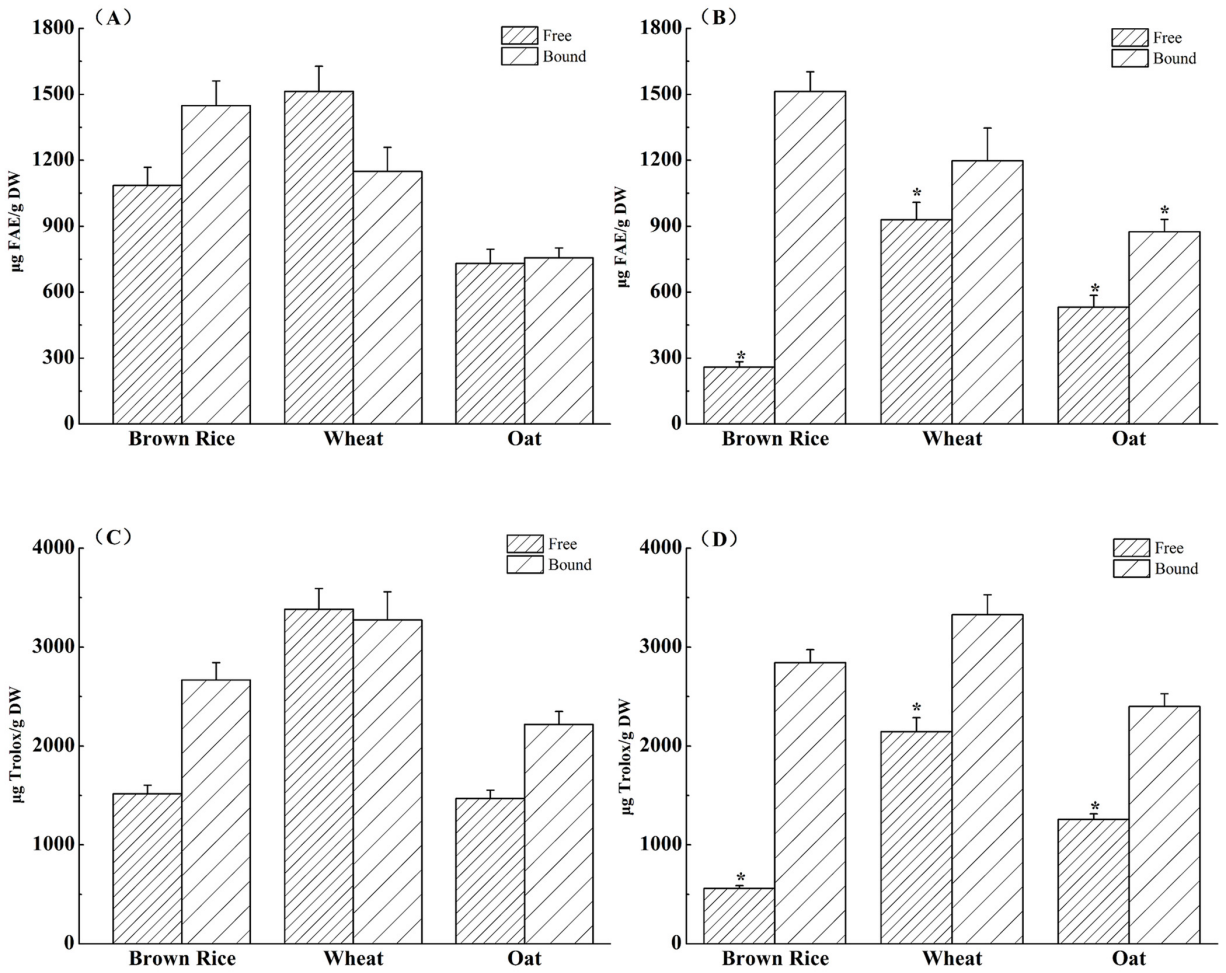
Total Phenolic content and total antioxidant activity of raw (A, C) and processed (B, D) cereal grains. * Significantly (*p* < 0.05) different from raw cereals.

The content of free phenolics in wheat was 1513.19 μg FAE/g DW, followed by brown rice (1085.99 FAE/g DW) and oat (730.27 μg FAE/g DW) (Fig 1). Moreover, the bound phenolics in brown rice was the highest (1448.72 μg FAE/g DW), and it accounted for 57.16% of the total phenolic content. After IECT, a significant loss of the free phenolics in brown rice (76.18%), wheat (38.63%) and oat (27.16%) was observed, which was due to the simultaneous actions of heat, pressure, and shear during IECT. On the other hand, the bound phenolics of brown rice and oat were increased by 4.47% and 15.60%, respectively. Some reports showed that processing could cause damage of cell structures and facilitate the release of bioactive compounds from the matrix, thus enhance the extractability of bound phenolics in the materials [15–16]. However, the bound phenolics of wheat were almost unchanged after processing, which may be due to the difference of cereal matrix.

The main contributor of antioxidant activity in raw and processed cereals was in bound form, and it was increased by IECT. However, the total antioxidant activity of free form for brown rice, wheat, and oat were significantly decreased by 63.06%, 36.55%, 14.39%, respectively. Similar results were also observed by Sharma et al. [17], who stated that processing had greater effect on free antioxidants than those bound forms in the food matrix. Herein, the changes of antioxidant activity were similar to that of phenolic content, suggesting that the phenolic compounds played a major part in antioxidant activity of cereals.

In summary, the total phenolic content and total antioxidant activity of free form in cereals was significantly decreased, while the bound form was increased after IECT. Additionally, the extent of IECT impact varied with different cereals, which were due to that the cereal matrix and phenolic acids profile of cereals may also affect the phenolic content and antioxidant activity during IECT.

### Effect of IECT on Phenolic Acids of Cereals

**Fig 2:**
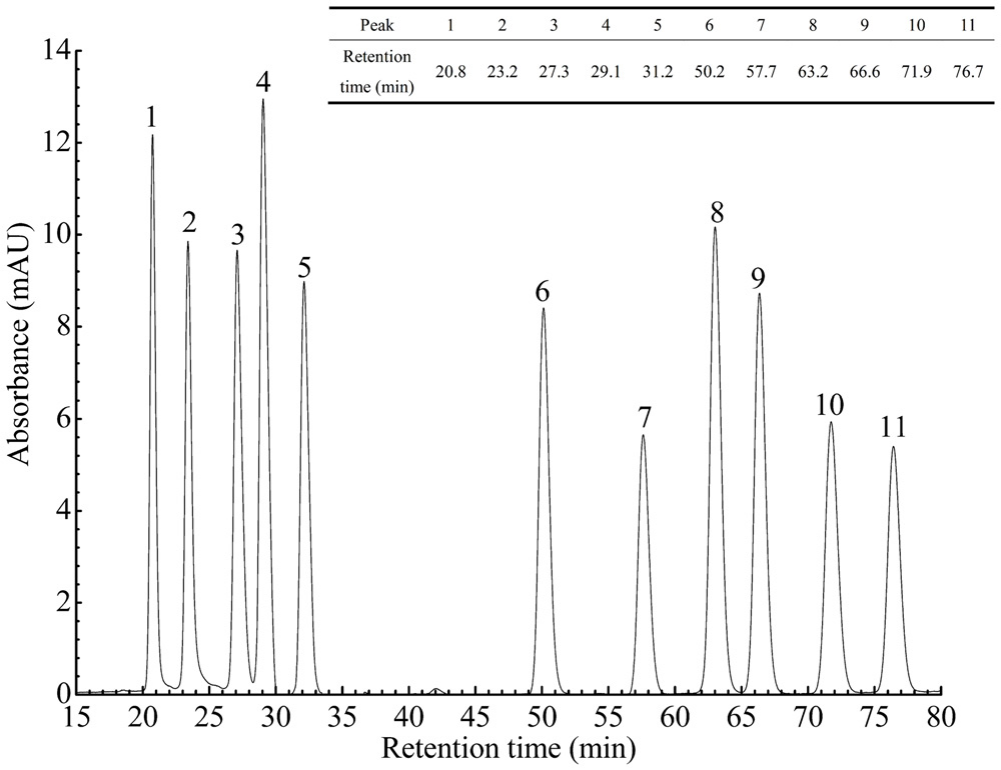
HPLC chromatogram of phenolic acid standards. 1: *p*-hydroxybenzoic acid; 2: chlorogenic acid; 3: vanillic acid; 4: *trans*-caffeic acid; 5: syringic acid; 6: *trans*-*p*-coumaric acid; 7: *cis*-*p*-coumaric acid; 8: *trans*-ferulic acid; 9: *trans*-sinapic acid; 10: *cis*-ferulic acid; 11: *cis*-sinapic acid.

Phenolic acid is the most common phenolic compound in cereals, and two major groups of them are commonly found, namely hydroxybenzoic acid and hydroxycinnamic acid derivatives [1]. The HPLC chromatogram of 11 standard phenolic acids is shown in Fig 2. All the chromatograms of the three cereals showed these peaks, which revealed that 11 phenolic acids existed in these cereals. The content of phenolic acids of different cereal samples is summarized in Table 1. The major phenolic acids detected in raw and processed cereals were *p*-hydroxybenzoic, chlorogenic, vanillic, caffeic, syringic, *p*-coumaric, ferulic and sinapic acids. It was worth noting that a large amount of *cis*-phenolic acids were observed in cereals and mostly in bound form, which was usually ignored in the previous studies. They may possess the same antioxidant activities or even exert higher physiological activities than *trans*-phenolic acids in many aspects [18–19]. Furthermore, more than 90% of the phenolic acids existed in bound form. It was widely reported that phenolic acids in cereals were mainly in bound form, involved in linkages of ester, ether, or acetal bonds to cellulose, proteins or lignin [1, 11, 20].

**Table 1:** The content (μg/g) of phenolic acids in raw and processed cereals determined by HPLC.

Phenolic acid	Sample	Raw cereal		IECT	
		Free	Bound	Free	Bound
*p*-hydroxybenzoic acid	Brown rice	1.08±0.03	2.87±0.05	0.67±0.06 *	5.10±0.06 *
	Wheat	39.43±0.41	4.66±1.01	15.68±1.28 *	9.59±2.92
	Oat	1.20±0.04	2.80±0.51	1.70±0.16 *	6.85±0.03 *
chlorogenic acid	Brown rice	0.48±0.01	6.78±0.40	0.12±0.02 *	9.63±0.01 *
	Wheat	1.06±0.84	6.40±1.01	−	9.05±0.97 *
	Oat	3.70±0.19	15.64±0.15	2.01±0.15 *	17.40±0.06 *
vanillic acid	Brown rice	1.18±0.03	1.84±0.03	0.70±0.04 *	2.89±0.04 *
	Wheat	2.42±0.19	3.76±0.47	2.33±0.11	4.22±0.12
	Oat	2.47±0.02	4.76±0.01	2.80±0.09 *	8.02±0.31 *
*trans*-caffeic acid	Brown rice	0.23±0.05	0.76±0.11	0.21±0.04	2.42±0.03 *
	Wheat	2.20±0.18	29.63±0.12	2.04±0.06	23.59±0.83 *
	Oat	3.72±0.07	11.88±0.84	2.96±0.14 *	21.73±1.40 *
syringic acid	Brown rice	0.28±0.11	4.20±0.19	0.50±0.01 *	3.99±0.22
	Wheat	0.62±0.02	1.84±0.14	1.22±0.06 *	3.59±0.24 *
	Oat	2.03±0.01	4.28±0.08	2.60±0.11 *	8.29±0.15 *
*trans*-*p*-coumaric acid	Brown rice	1.21±0.03	54.38±2.62	0.81±0.01 *	54.03±2.09
	Wheat	0.60±0.01	34.46±3.38	0.79±0.04 *	31.79±2.68
	Oat	0.49±0.01	16.76±1.81	0.42±0.06	15.24±2.21
*cis*-*p*-coumaric acid	Brown rice	−	5.53±0.34	0.13±0.01 *	4.32±0.79
	Wheat	−	7.18±0.01	−	5.27±1.14 *
	Oat	−	3.50±0.55	−	3.35±0.37
*trans*-ferulic acid	Brown rice	2.96±0.09	258.90±6.94	1.43±0.02 *	267.17±6.10
	Wheat	2.25±0.03	389.74±10.94	3.19±0.20 *	431.30±14.81 *
	Oat	2.38±0.05	162.28±4.60	2.22±0.12	230.81±7.95 *
*trans*-sinapic acid	Brown rice	−	5.46±0.76	0.19±0.01 *	17.24±0.36 *
	Wheat	−	7.26±0.38	0.47±0.12 *	13.56±0.43 *
	Oat	1.35±0.03	5.28±0.80	1.69±0.02 *	22.10±0.87 *
*cis*-ferulic acid	Brown rice	0.41±0.03	50.02±4.74	0.28±0.06 *	44.45±6.83
	Wheat	0.29±0.02	68.10±0.18	0.38±0.04 *	68.37±12.72
	Oat	0.46±0.01	27.53±3.04	0.46±0.03	41.93±3.60 *
*cis*-sinapic acid	Brown rice	−	−	−	4.71±0.40 *
	Wheat	0.63±0.01	1.78±0.12	0.81±0.05 *	3.16±0.39 *
	Oat	0.49±0.03	−	0.54±0.10	4.06±0.32 *
Total phenolic acids	Brown rice	7.86±0.30	390.75±5.59	5.04±0.08 *	415.94±0.39 *
	Wheat	49.50±0.85	554.81±14.49	26.90±1.89 *	603.50±4.66 *
	Oat	18.31±0.38	254.73±2.41	17.41±0.47	379.79±6.84 *

Values are means ± SD for triplicates.

* Significantly (*p* < 0.05) different from raw cereals.

The dominant phenolic acid (sum of free and bound forms) in raw brown rice was *trans*-ferulic acid (261.86 μg/g), followed by *trans*-*p*-coumaric acid (55.59 μg/g) (Table 1), which was in agreement with the previous reports [11, 21–22]. Compared to brown rice, wheat contained more phenolic acids both in free (6.30-fold) and bound (1.42-fold) forms. The total phenolic acids (sum of free and bound forms) of wheat (604.31 μg/g) was higher than that of brown rice (398.61 μg/g), which was consistent with previous studies [23]. Besides, *tans*-ferulic acid (391.99 μg/g) was the most abundant in wheat, followed by *cis*-ferulic acid (68.39 μg/g), which was different from that of brown rice. Particularly, free *p*-hydroxybenzoic acid was abundant in the present study and constituted 79.66% of the total free phenolic acids in wheat. The dominant phenolic acid in oat was *tans*-ferulic acid (164.66 μg/g) followed by *cis*-ferulic acid (27.99 μg/g). The total phenolic acids of oat were 273.04 μg/g, which were lower than that of brown rice (398.61 μg/g) and wheat (604.31 μg/g). However, oat had more free phenolic acids (18.31 μg/g) than brown rice (7.86 μg/g).

The IECT greatly affected the composition of both free and bound phenolic acids in cereals. Specifically, free chlorogenic acid in wheat, *cis*-*p*-coumaric acid in brown rice, *trans*-sinapic acid in brown rice and wheat, and the bound form of *cis*-sinapic acid in oat were greatly affected by IECT. Besides, the content of most phenolic acids in cereals was significantly different after IECT. The total free phenolic acids was significantly decreased in brown rice (35.88%) and wheat (45.66%) after IECT, but the total free phenolic acids in oat was less affected by IECT. In addition, the total bound phenolic acids of brown rice, wheat, and oat were significantly increased by 6.45%, 8.78%, and 49.10% after IECT. IECT could inevitably lead to a decrease of free phenolic acids because of decomposition caused by the high temperature. Besides, the increased polymerization of free phenolic acids during IECT could decrease their extractability [24]. Moreover, decarboxylation of free phenolic acids during IECT may, in turn, promote polymerization of phenolic acids, leading to reduced extractability [25]. Additionally, bound phenolic acids may release from the cell walls and could be transformed to free phenolic acids during IECT. However, changes in the organizational structure of the extruded cereals may also lead to the increases in extractability of bound phenolic acids. Furthermore, the effects of IECT on phenolic acids in brown rice, wheat, and oat were different, which was due to the great difference of cereal matrix. Therefore, cereal matrix and the sensitivity of free and bound phenolic acids during IECT greatly affected the profile of phenolic acids of cereals.

### Effect of IECT on Bioaccessibility of Phenolics and Antioxidant Compounds of Cereals

To exert bioactivity, phenolic compounds have to be firstly bioaccessible, i.e., released from the food matrix and solubilised after digestion. Therefore, an *in vitro* gastrointestinal model was used in this study to mimic the *in vivo* physiological environment. The content of bioaccessible phenolics of brown rice, wheat, and oat were 528.99, 308.83, and 443.44 μg FAE/g DW, respectively. Besides, the bioaccessible antioxidant compounds of brown rice, wheat, and oat were 1260.94, 635.77, and 681.05 μg Trolox/g DW, respectively. Even though cereals possessed appreciable amounts of phenolics and antioxidant compounds, the amount of bioaccessible compounds was considerably low. It was due to that the phenolics mainly existed in covalently bound form in cereals. Therefore, they could survive the gastrointestinal digestion to reach the colon. In addition, although wheat possessed the highest total phenolic content and total antioxidant activity (Fig 1), the bioaccessible phenolics and antioxidant compounds were lower than that of brown rice and oat (Table 2). It indicated that cereals with the most abundant phenolics were not necessarily those with the highest bioccessibility. The cereal matrix seemed to be a crucial factor in their digestibility and stabilty in the digestion, which influenced the bioaccessibility.

**Table 2:** The content of bioaccessible phenolics and antioxidant compounds of raw and processed cereals.

Cereal grains		Bioaccessible phenolic content (μg FAE/g DW)	Bioaccessibility of phenolics (%)	Bioaccessible antioxidants content (μg Trolox/g DW)	Bioaccessibility of antioxidants (%)
Brown rice	Raw cereal	528.99±41.05	20.87	1260.94±76.58	30.15
	IECT	364.54±38.82 *	20.57	992.49±52.96 *	29.16
Wheat	Raw cereal	308.83±32.62	11.60	635.77±64.88	9.55
	IECT	265.24±19.38	12.48	606.70±53.23	11.08
Oat	Raw cereal	443.44±34.59	29.83	681.05±81.14	18.47
	IECT	306.20±38.94 *	21.78	626.12±47.98	17.12

Values are means ± SD for triplicates.

* Significantly (*p* < 0.05) different from raw cereals.

After IECT, the bioaccessible phenolics of brown rice and oat were significantly decreased by 31.09% and 30.95%, which was caused by the decrease of free phenolics. However, the bioaccessible phenolics and antioxidant compounds of wheat were almost unchanged after IECT, which may be due to the difference of cereal matrix.

### Effect of IECT on Bioaccessibility of Phenolic Acids of Cereals

The profile of bioaccessible phenolic acids of raw cereals is shown in Table 3. Not all kinds of phenolic acids were bioaccessible. Among the 11 identified phenolic acids, there were only 7, 7, and 6 phenolic acids bioaccessible in brown rice, wheat, and oat, respectively. The dominant bioaccessible phenolic acids of brown rice, wheat, and oat were *trans*-ferulic acid, *p*-hydroxybenzoic acid, and syringic acid, respectively. In addition, the content of bioaccessible phenolic acids of cereals was considerably low with values in the rang of 0.08 to 2.33 μg/g, and the total bioaccessible phenolic acids of brown rice, wheat, and oat were 1.52, 3.59, 1.36 μg/g, respectively. The total bioaccessible phenolic acids of wheat were higher than those of brown rice and oat, which was probably ascribed to that a considerable amount of phenolic acids of wheat existed in free form.

**Table 3:** The content (μg/g) of phenolic acids in dialysate samples of raw and processed cereals determined by HPLC.

Phenolic acids	Sample	Raw cereal	IECT
*p*-hydroxybenzoic acid	Rice	0.22±0.01	0.18±0.01
	Wheat	2.33±0.03	0.94±0.08
	Oat	0.26±0.01	0.26±0.01
chlorogenic acid	Rice	−	−
	Wheat	−	−
	Oat	−	−
vanillic acid	Rice	0.29±0.01	0.19±0.01
	Wheat	0.28±0.01	0.25±0.01
	Oat	0.33±0.03	0.16±0.03
*trans*-caffeic acid	Rice	−	−
	Wheat	0.18±0.06	0.14±0.01
	Oat	−	−
syringic acid	Rice	0.11±0.01	0.11±0.01
	Wheat	0.17±0.02	0.21±0.01
	Oat	0.34±0.01	0.09±0.01
*trans*-*p*-coumaric acid	Rice	0.23±0.01	0.21±0.01
	Wheat	0.12±0.01	0.12±0.01
	Oat	0.10±0.01	−
*cis*-*p*-coumaric acid	Rice	0.08±0.01	0.07±0.01
	Wheat	−	−
	Oat	−	−
*trans*-ferulic acid	Rice	0.41±0.01	0.37±0.01
	Wheat	0.22±0.04	0.33±0.05
	Oat	0.17±0.03	−
*trans*-sinapic acid	Rice	−	−
	Wheat	−	−
	Oat	−	−
*cis*-ferulic acid	Rice	0.18±0.01	0.15±0.01
	Wheat	0.12±0.02	0.13±0.02
	Oat	0.15±0.01	−
Total phenolic acids	Rice	1.52±0.02	1.29±0.03
	Wheat	3.59±0.10	2.11±0.12
	Oat	1.36±0.01	0.52±0.03

Values are means ± SD for triplicates.

IECT had no significant effect on the composition of bioaccessible phenolic acids of brown rice and wheat. However, the *trans*-*p*-coumaric acid, *trans*-ferulic acid, and *cis*-ferulic acid in oat were not bioaccessible after IECT. In addition, most of the bioaccessible phenolic acids were decreased by IECT, and the total bioaccessible phenolic acids in brown rice (1.52 μg/g), wheat (3.59μg/g), and oat (1.36 μg/g) were decreased to 1.29, 2.11, 0.52 μg/g, respectively.

Even thouth phenolic acid was the most common phenolic compound in cereals, the bioaccessibility of phenolic acids (< 1%) was considerably low both in raw and processed cereals. This finding was consistent with previous studies on the bioavailability of ferulic acid in cereal bran and aleurone [26–27]. However, the poor bioaccessibility of phenolic acids in cereals (< 1%) seemed to contradict the bioaccessibility of phenolics (12.48%-21.78%) and antioxidant compounds (11.08%-29.16%) (Table 2). The primary reason for this large discrepancy was that many of the compounds presented in dialysate samples, except phenolic acids, were unknown. Therefore, the phenolic acids accounted for a small portion of bioaccessible phenolics in cereals. It indicated that phenolic acids slightly contributed to the human health after gastrointestinal digestion, although it is the major phenolics in cereals.

## Conclusions

This work demonstrated that the IECT greatly affected the total phenolic content, total antioxidant activity, and phenolic acid of cereals, with the effect depending on cereal matrix and the sensitivity of free and bound phenolics. In addition, cereals with the most abundant phenolics were not necessarily those with the highest bioccessibility. IECT significantly decreased the bioaccessible phenolics of brown rice and oat, while it had minimal effect on the bioaccessible phenolics of wheat. Furthermore, bioaccessible phenolic acids of raw and processed cereals were considerably low, and they slightly contributed to the bioaccessible phenolics. Therefore, the contributions of phenolic acids to the human health after gastrointestinal digestion were extremely limitted. Further research was needed to identify and quantify the bioaccessible antioxidant compounds except phenolic acids. The effects of IECT on the bioavailability of phenolics of cereals *in vivo* were also required.

## Supporting Information

S1 Experimental Data (XLSX)
